# Novel and unusual genes for nitrogen and metal cycling in *Planctomycetota*- and KSB1-affiliated metagenome-assembled genomes reconstructed from a marine subsea tunnel

**DOI:** 10.1093/femsle/fnad049

**Published:** 2023-06-08

**Authors:** Carolina Suarez, Thomas Hackl, Britt-Marie Wilen, Frank Persson, Per Hagelia, Mike S M Jetten, Paula Dalcin Martins

**Affiliations:** Division of Water Resources Engineering, Faculty of Engineering LTH, Lund University, Lund 221 00, Sweden; Microbial Ecology Cluster, GELIFES, University of Groningen, Groningen 9747 AG, Netherlands; Division of Water Environment Technology, Department of Architecture and Civil Engineering, Chalmers University of Technology, Gothenburg 412 96, Sweden; Division of Water Environment Technology, Department of Architecture and Civil Engineering, Chalmers University of Technology, Gothenburg 412 96, Sweden; Construction Division, The Norwegian Public Roads, Administration, Oslo 0667, Norway; Department of Microbiology, RIBES, Radboud University, Nijmegen 6525 AJ, Netherlands; Microbial Ecology Cluster, GELIFES, University of Groningen, Groningen 9747 AG, Netherlands

**Keywords:** nitrogen cycling, anammox bacteria, nitrite oxidizers, metal cycling, *Planctomycetota*, KSB1 phylum

## Abstract

The Oslofjord subsea road tunnel is a unique environment in which the typically anoxic marine deep subsurface is exposed to oxygen. Concrete biodeterioration and steel corrosion in the tunnel have been linked to the growth of iron- and manganese-oxidizing biofilms in areas of saline water seepage. Surprisingly, previous 16S rRNA gene surveys of biofilm samples revealed microbial communities dominated by sequences affiliated with nitrogen-cycling microorganisms. This study aimed to identify microbial genomes with metabolic potential for novel nitrogen- and metal-cycling reactions, representing biofilm microorganisms that could link these cycles and play a role in concrete biodeterioration. We reconstructed 33 abundant, novel metagenome-assembled genomes (MAGs) affiliated with the phylum *Planctomycetota* and the candidate phylum KSB1. We identified novel and unusual genes and gene clusters in these MAGs related to anaerobic ammonium oxidation, nitrite oxidation, and other nitrogen-cycling reactions. Additionally, 26 of 33 MAGs also had the potential for iron, manganese, and arsenite cycling, suggesting that bacteria represented by these genomes might couple these reactions. Our results expand the diversity of microorganisms putatively involved in nitrogen and metal cycling, and contribute to our understanding of potential biofilm impacts on built infrastructure.

## Teaser

We identified genes involved in anaerobic ammonium oxidation, nitrite oxidation, and iron and manganese cycling in bacterial genomes reconstructed from concrete-degrading biofilms in the Oslofjord subsea road tunnel.

## Introduction

The marine deep biosphere comprises a significant part of life on Earth (Bar-On et al. 2018), but it is still largely unexplored. The Oslofjord subsea tunnel in Norway is a unique environment in which the marine deep subsurface, typically comprised of anoxic sediments and jointed rock mass, is exposed to oxygen in the tunnel. This subsea road tunnel has a maximum depth of 134 m below sea level and is covered by sprayed concrete, employed directly onto the rock mass, reinforced with steel fibers for rock support of the tunnel structure. However, cracks in the bedrock allow seepage of saline water from the overlying water column through the bedrock and across the sprayed concrete layer. In areas of the tunnel with water seepage, a biofilm has developed on the sprayed concrete surface, causing biodeterioration of the concrete with associated steel fiber corrosion (Karačić et al. 2018). The biofilm consists of an outer orange to brown layer, rich in amorphous iron hydroxide (ferrihydrite), and an inner black layer, rich in manganese oxide biominerals (Na-buserite, todorokite, and birnessite) (Hagelia 2007, 2011). Reduction of these iron hydroxides, manganese oxides and, additionally, sulfate, has been detected in some biofilms (Hagelia 2011, Karačić et al. 2018).

Biotic and abiotic reactions within the biofilm lead to acidification of the saline water from pH 7.5–8 to 5.5–6.5 at low water flow rates (Hagelia 2011). A likely responsible mechanism for the acidification is microbial oxidation of Fe^2+^ and Mn^2+^ with oxygen, which, upon precipitation of Fe^3+^ and Mn^4+^ biominerals, releases H^+^ (Manahan 2000). However, these reactions can also occur at circumneutral pH (Emerson 2000). Additionally, the penetration of chloride and the deposition of Mn-oxides is known to cause pitting corrosion on steel (Dickinson et al. 1997, Olesen et al. 2001, Hagelia 2011). The acidic water causes deep disintegration and enhances the porosity of the cement paste matrix due to dissolution of portlandite and calcium silicate hydrate, leading to formation of carbonates, thaumasite sulfate attack and magnesium attack (Hagelia 2011, Karačić et al. 2018).

Based on these previous studies, metal-cycling microorganisms were expected to be abundant in biofilms. However, when the 16S rRNA gene diversity of biofilm samples collected from three tunnel areas was analyzed (Karačić et al. 2018), microbial communities were surprisingly dominated by putative nitrogen-cycling members: the most abundant amplicon sequence variant (ASV) across 64 biofilm samples was affiliated with the ammonium-oxidizing archaeon *Nitrosopumilus*. Other highly abundant ASVs were affiliated with betaproteobacterial ammonium-oxidizing *Nitrosomonadaceae*, marine nitrite-oxidizing *Nitrospina*, nitrifying *Nitrospira*, and marine anaerobic ammonium-oxidizing (anammox) *Candidatus* Scalindua (Karačić et al. 2018). Additionally, a follow-up metagenomics study identified in these biofilms a novel family of anammox bacteria named *Ca*. Anammoxibacteraceae (Suarez et al. 2022). These results suggested that novel microorganisms enriched in Oslofjord tunnel biofilms could perform metabolic reactions linking nitrogen and metal biogeochemical cycling.

Here, we reconstructed metagenome-assembled genomes (MAGs) from Oslofjord tunnel biofilm samples representing abundant community members affiliated with novel taxa. This study aimed to identify the metabolic potential for novel nitrogen- and metal-cycling reactions, thus expanding the known diversity of microorganisms with the potential of linking these cycles. This resulted in the selection of 33 MAGs affiliated with the phylum *Planctomycetota* and candidate phylum KSB1, which were interrogated with respect to their potential biogeochemical repertoire. Typically, both phyla have broad metabolic potential and are implicated in heterotrophic lifestyles. *Planctomycetota* are frequently described as extremely diverse bacteria with unusual cell biology and aerobic or facultative anaerobic, chemoheterotrophic metabolism (Elshahed et al. 2007, Spring et al. 2018, Wiegand et al. 2018), with the exception of the anaerobic lithoautotrophic anammox bacteria (Kartal et al. 2012). Similarly, while no representatives of the candidate phylum KSB1 have been cultured to date, MAG analyses indicate that these microorganisms are likely involved in organic carbon degradation and fermentation in estuarine (Baker et al. 2015) and hydrothermal sediments (Dombrowski et al. 2017), harboring genes encoding multiple carbohydrate-active enzymes (López-Mondéjar et al. 2022) and potentially novel isopropanol dehydrogenases (Dalcin Martins et al. 2019).

In particular, we searched for both canonical and divergent marker genes involved in nitrogen cycling pathways. These included anaerobic ammonium oxidation via a reductive hydroxylamine oxidoreductase-encoding gene (*hao*) for nitrite reduction to nitric oxide (Ferousi et al. 2021), hydrazine synthase (*hzsABC*) for ammonium oxidation coupled to nitric oxide reduction, producing hydrazine (Dietl et al. 2015a), and hydrazine dehydrogenase (*hdh*), for hydrazine oxidation to dinitrogen gas (Maalcke et al. 2016). A gene encoding hydroxylamine oxidase (*hox*), with unknown physiological function but conserved in anammox bacteria (Kartal and Keltjens 2016), was included in our analyses. We also searched for genes in aerobic (complete) nitrification (van Kessel et al. 2015) via ammonium monooxygenase (*amoABC*), for ammonium oxidation to hydroxylamine, hydroxylamine oxidoreductase (*hao*), for hydroxylamine oxidation to nitrite, and nitrite oxidoreductase (*nxrABC*) for nitrite oxidation to nitrate (Daims et al. 2016a). Genes in the denitrification pathway (Philippot 2002) comprised both membrane-bound (*narGHI*) and periplasmic (*napAB*) nitrate reductases for nitrate conversion to nitrite, nitrite reductase for nitrite reduction to nitric oxide (*nirK* and *nirS*) or to ammonium (*nrfAH*), nitric oxide reductase for nitric oxide conversion to nitrous oxide (*norB*), and nitrous oxide reductase for the last step in denitrification, nitrous oxide reduction to dinitrogen gas (*nosZ*).

Additionally, we searched for genes encoding manganese- and iron-cycling proteins: the manganese oxidase-encoding genes *mnxG* and *mcoA* (Geszvain et al. 2013), *moxA* (Ridge et al. 2007), and *cotA* (Su et al. 2013), the iron oxidase-encoding gene *cyc2* (McAllister et al. 2020), and several genes encoding iron reductase complexes (Garber et al. 2020), such as (outer membrane) *c*-type cytochromes (Omc) and porin-cytochrome *c* (PCC) complexes. Microorganisms that reduce iron can frequently reduce manganese, in some instances using the same proteins, such as OmcS and OmcZ (Richter et al. 2012) and MtrCAB (Szeinbaum et al. 2014). Therefore, in this study, MAGs with potential for iron reduction could also represent microorganisms capable of reducing manganese, and therefore are referred to as presenting general metal-cycling potential.

## Materials and methods

The Oslofjord subsea tunnel is part of road E134 near Drøbak in Norway (59.66 472 N, 10.61 306 E). Biofilms in two areas of the tunnel wall, referred to as pump station and test site, were sampled four times in total in 2016, 2017, 2019, and 2020. Biofilm sampling, DNA extractions, and shotgun metagenomic sequencing were performed as previously described (Karačić et al. 2018, Suarez et al. 2022). Briefly, Illumina NovaSeq6000 sequencing generated 150 bp paired-end reads, which were normalized to 100 × coverage using BBNorm in the BBTools package 38.61b (https://sourceforge.net/projects/bbmap) and co-assembled with Megahit 1.2.9 (Li et al. 2015). Reads were mapped to the assembly with Bowtie v2.3.5.1 (Langmead and Salzberg 2012), which was binned with MetaBAT2 v2.15 (Kang et al. 2019) and BinSanity v0.5.3 (Graham et al. 2017). MAGs were dereplicated with DASTool v1.1.2 (Sieber et al. 2018) and retained only if less than 10% contaminated and more than 50% complete, as determined with CheckM (Parks et al. 2015). Additionally, MAGs were inspected for chimerism and contamination with GUNC v1.05 (Orakov et al. 2021). MAGs were classified with GTDB-Tk v1.5.0 (Chaumeil et al. 2019) with the GTDB 07-RS207 taxonomy (Parks et al. 2020), and their relative abundances were calculated with coverM v0.6.1 (https://github.com/wwood/CoverM) with the relative_abundance parameter in genome mode using BWA-MEM (Li 2013). Metagenome reads and MAGs from the Oslofjord tunnel biofilms are publicly available in the NCBI BioProject PRJNA755678.

MAGs were annotated with DRAM v1.0 (Shaffer et al. 2020) with default options, except -min_contig_size 1000, and most genes of interest were searched in annotation files. Additionally, some genes were identified via complementary methods: genes encoding proteins involved in anammox metabolism were searched both via annotation files and via blastp analyses using previously identified reference sequences from *Ca*. Kuenenia stuttgartiensis (de Almeida et al. 2016, Kartal and Keltjens 2016), and iron cycling-related genes were detected with FeGenie (Garber et al. 2020). Phylogenetic trees were built with FastTree v2.1.10 (Price et al. 2010) and visualized in iToL v6 (Letunic and Bork 2021), with the exception of the tree containing UBA1845 MAGs from this study and reference genomes, which was built with IQ-TREE v2.2.0 (Minh et al. 2020) from an alignment of 74 single copy genes done with GToTree v1.7.00 (Lee 2019). Heat maps were generated in RStudio v4.2.1 using the vegan package v2.6–4 (Oksanen et al. 2019). Gene clusters were identified and visualized in R with the standard gggenomes workflow (https://github.com/thackl/gggenomes). Divergent sequence similarity analyses were performed with HHpred (https://toolkit.tuebingen.mpg.de/tools/hhpred). All figures were edited in Adobe Illustrator.

## Results and discussion

### *Planctomycetota*- and KBS1-affiliated MAGs were abundant across biofilm samples.

**Figure 1.**
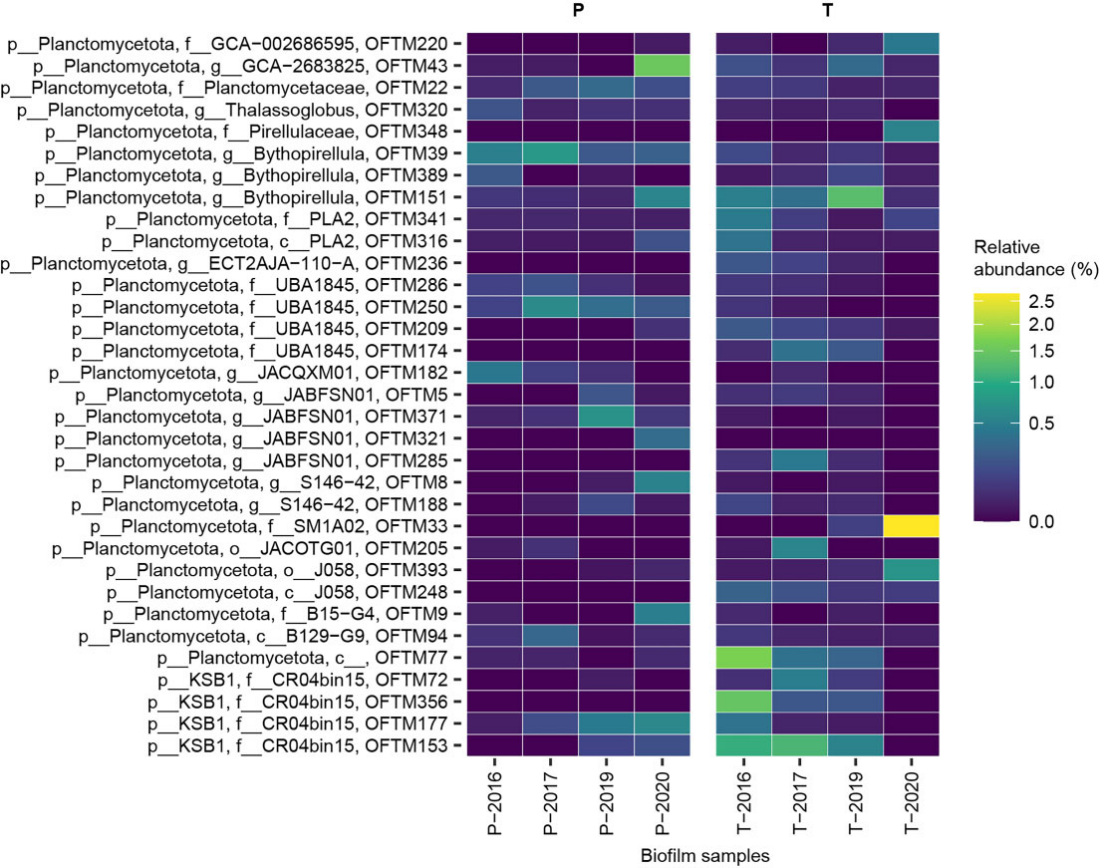
Relative abundance of MAGs in pump station (P) and test site (T) samples collected from the Oslofjord tunnel in four years (2016–2020). Values are provided in Supplementary Table 1.

We analyzed our MAG dataset (NCBI BioProject PRJNA755678) for metabolic potential regarding novel nitrogen- and metal-cycling reactions. Upon MAG inspection for accuracy of assembly and binning, 33 MAGs were selected for this study, of which 24 had high quality (>90% completeness and < 5% contamination) and 9 had medium quality (here, >75% completeness and < 8% contamination) (Bowers et al. 2017). Individually, the MAGs selected for this study reached up to 2.5% of relative abundance in the biofilm community, summing 1.7%–7.6% of the community across biofilm samples (Fig. 1), which were collected in four instances between 2016 and 2020 from two tunnel areas: the pump site, with sprayed concrete since 1999 for permanent rock support, and the test site, with sprayed concrete since 2010 to test concrete durability (Hagelia 2011). The retrieved MAGs could not be easily classified beyond the phylum level: all four of the candidate phylum KSB1-affiliated MAGs belonged to the putative family ‘CR04bin15’. Furthermore, only 6 of 29 MAGs within the phylum *Planctomycetota* could be classified beyond the putative family level (Supplementary Table 1). Next, based on taxonomic novelty, we focused on searching for genes involved in nitrogen and metal cycling.

### Genes with sequence similarity to hydrazine synthase subunits were present in several *phycisphaerae* MAGs.

Anaerobic ammonium oxidation (anammox) is an important process in the nitrogen cycle and is catalyzed by the enzyme hydrazine synthase, encoded by three genes (*hzsABC*) used as markers for this metabolism (Harhangi et al. 2012). We identified 21 genes that had blastp hits with a bitscore >40 to *hzsABC* from *Ca*. Kuenenia stuttgartiensis across 17 genomes in this study (Supplemental Table 1), hereafter referred to as *hzs*-like genes. While a minimum bitscore value of 60 is the default used for DRAM annotations (Shaffer et al. 2020), we used this low bitscore threshold to allow for the identification of divergent sequences.

**Figure 2.**
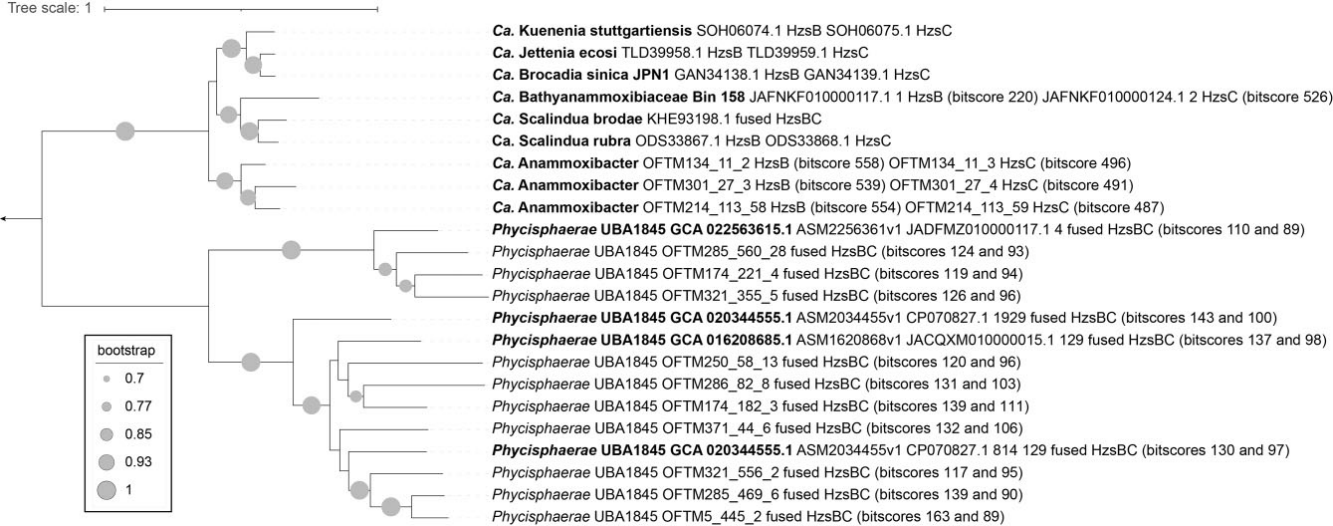
Phylogenetic tree of *hzsB* and *hzsC*(-like) genes (concatenated protein sequences unless indicated as fused genes. Bold indicates reference sequences retrieved from NCBI with respective accession numbers, while the other sequences were obtained from this study. Only sequences with an *hzsA* gene located upstream of *hzsBC* were included in the tree. Bitscore values were obtained from blastp hits (Supplementary Table 1) to *Ca*. Kuenenia stuttgartiensis HzsB and HzsC sequences, respectively, present in the tree. The tree was rooted in the *Brocadiales* (upper) clade.

**Figure 3.**
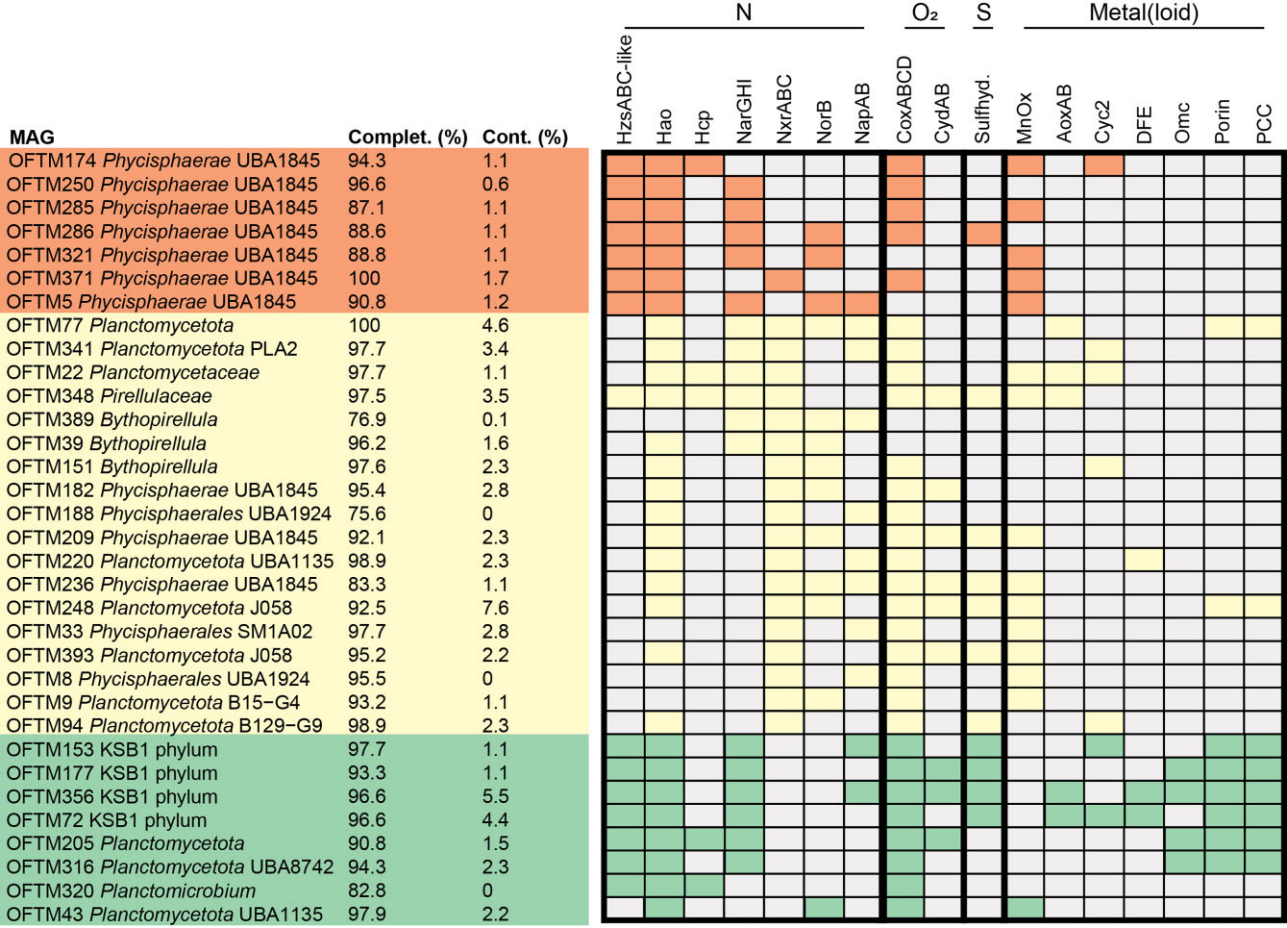
Summary of metabolic potential identified in MAGs in this study. MAGs representing organisms with potential for anammox metabolism are highlighted in orange, for nitrite oxidation in yellow, and for other reactions in nitrogen and metal cycling in green. The presence of genes encoding proteins involved in nitrogen (N), oxygen (O_2_), sulfur (S), and metals (iron and manganese) or metalloid (arsenic) cycling is indicated by the corresponding metabolic group colors, while the absence of genes is indicated by grey. Proteins are as follows: HzsABC-like, genes with sequence similarity to subunits of hydrazine synthase; Hao, hydroxylamine oxidoreductase; Hcp, hydroxylamine reductase; NarGHI, putative membrane-bound nitrate reductase; NxrABC, putative membrane-bound nitrite oxidoreductase; NorB, nitric oxide reductase; NapAB, periplasmic nitrate reductase; CoxABCD, low-affinity cytochrome *c* oxidase/oxygen reductase; CydAB, high-affinity cytochrome *bd* ubiquinol oxidase/oxygen reductase; Sulfhyd.; sulfhydrogenase/elemental sulfur reductase; MnOx, manganese oxidase; AoxAB; arsenite oxidase; Cyc2, iron oxidase; DFE, *Desulfovibrio ferrophilus*-like flavin-based extracellular electron transfer complex for iron reduction; Omc, outer membrane cytochrome *c* for iron reduction; porin, porin involved in iron reduction; PCC, porin-cytochrome *c* complex for iron reduction.

**Figure 4.**
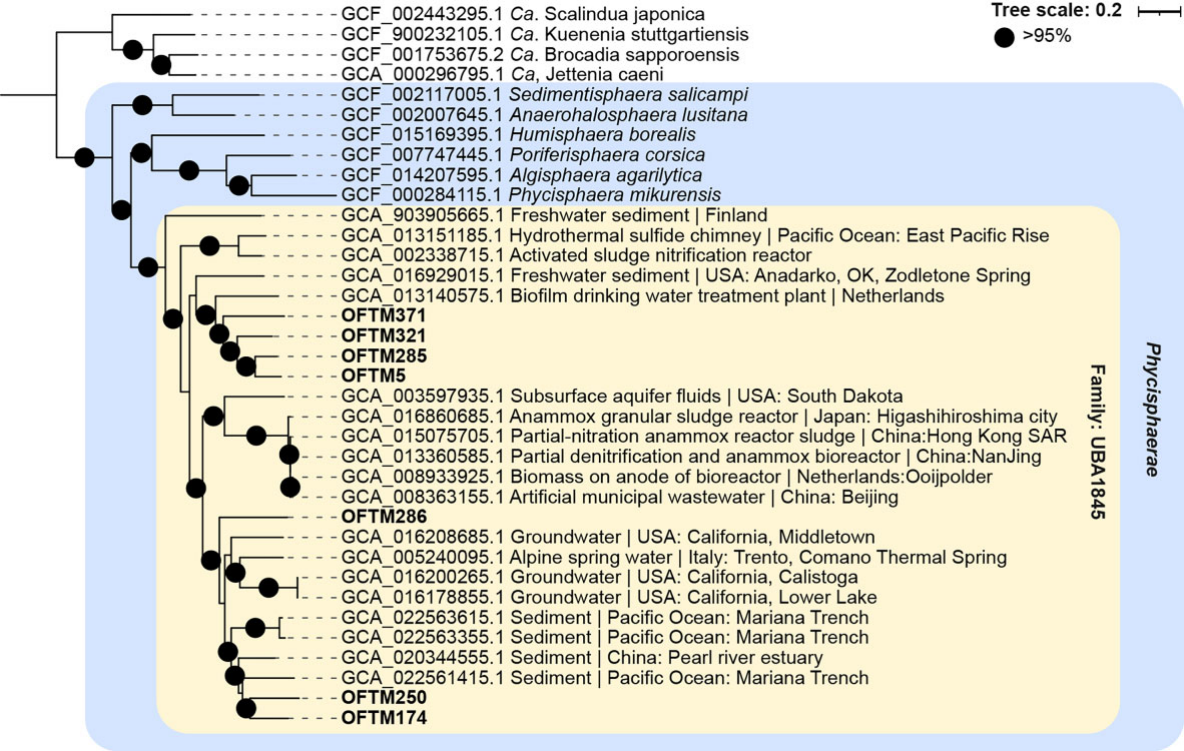
Biogeography of *Phycisphaerae* MAGs affiliated to the family UBA1845. The phylogenetic tree was built using an alignment of 74 single-copy genes (see methods) in MAGs retrieved from this study in combination with reference genomes retrieved from NCBI, as indicated by accession numbers. The order *Ca*. Brocadiales was used as outgroup. Black circles indicate branches with >95% ultrafast bootstrap support.

Several important genes potentially implicated in anammox metabolism were detected in seven MAGs affiliated with the class *Phycisphaerae*, within the putative family UBA1845: OFTM5, 174, 250, 285, 286, 321, and 371 (Figs. 2 and 3). These included 10 *hzsABC*-like genes with blastp-derived bitscore values ranging from 89 to 163 (in the annotation range) against *hzsABC* from *Ca*. Kuenenia stuttgartiensis (Fig. 2), as well as similar values when *hzsABC* sequences from *Ca*. Scalindua or *Ca*. Anammoxibacter were used. In these *Phycisphaerae* MAGs, *hzsB*- and *hzsC*-like genes were fused, as it has been observed in marine anammox *Ca*. Scalindua species (van de Vossenberg et al. 2013a, Dietl et al. 2015b), and had an *hzsA*-like gene encoded immediately upstream (Supplementary Table 1, Fig. 2). Similarly, we found *hzsABC*-like genes in three reference genomes (GCA_016 208 685.1, GCA_020 344 555.1 and GCA_022 563 615.1) affiliated with *Phycisphaerae* UBA1845, with *hzsA* immediately upstream of fused *hzsBC*-like subunits (Fig. 2). Additionally, we identified in these MAGs genes annotated as hydroxylamine oxidoreductases (*hao* and, only in OFTM5, also *hox*), nitrate/nitrite oxidoreductases (*narGHI* or *nxrABC*), R/b complex genes, ETM subunit 1 and 2-encoding genes, and other nitrogen cycle-related genes (Fig. 3 for a summary and Supplementary Table 1 for each gene annotation in each MAG). However, no hydrazine dehydrogenase- or nitrite reductase-encoding genes (*hdh, nirK*, or *nirS*) were identified in any genomes from this study. Furthermore, genes encoding subunits of oxygen reductases were detected in five of these seven MAGs, and genes encoding a nitric oxide reductase, periplasmic nitrate reductase, manganese, and iron oxidases were prevalent in *Phycisphaerae* genomes (Fig. 3). Analyses of reference genomes related to *Phycisphaerae* UBA1845 MAGs in our study indicated that these microorganisms are present in marine sediments and groundwater, as well as in wastewater and drinking water treatment plants (Fig. 4).

Based on these results, we hypothesize that these seven *Phycisphaerae* MAGs within the family UBA 1845 could represent novel anammox bacteria outside the order ‘*Ca*. Brocadiales’, which holds all currently described and hypothesized anammox taxa (Kartal et al. 2012, Suarez et al. 2022, Zhao et al. 2022), requiring future experimental validation by enrichment cultures and ^15^N isotope studies. The missing hydrazine dehydrogenase-encoding gene of the new MAGs could be too divergent to be detected based on sequence similarity or, alternatively, the identified hydroxylamine oxidoreductase could be involved in hydrazine oxidation to dinitrogen gas, an activity previously shown *in vitro* in *Ca*. Kuenenia stuttgartiensis (Maalcke et al. 2016), relying on a cross-linked active site heme (REF). Oxygen reductase genes present in these genomes might support the function of oxygen tolerance or detoxification, which has been recently described in anammox bacteria in bioreactors (Yang et al. 2022) and aquifer ecosystems (Mosley et al. 2022). Furthermore, MAGs comprising a novel clade II group of *Ca*. Brocadiae, likely anammox bacteria, were reconstructed from oxygenated aquifer samples and also lacked a hydrazine dehydrogenase-encoding gene (Mosley et al. 2022), as in our study. Finally, nitrate-dependent iron oxidation has been reported in *Ca*. Brocadia and *Ca*. Scalindua enrichment cultures (Oshiki et al. 2013), and metal oxide respiration has been described in *Ca*. Kuenenia stuttgartiensis, *Ca*. Brocadia, and *Ca*. Scalindua species (van de Vossenberg et al. 2013b, Strous et al. 2006, Oshiki et al. 2016), supporting the potential for metal-cycling metabolism detected in these *Phycisphaerae* MAGs that could represent novel anammox bacteria. Other MAGs in this study were not considered to represent potentially novel anammox because *hzsABC*-like genes in these MAGs had a low bitscore value (40–60) from blastp analyses using *Ca*. K. stuttgartiensis reference sequences, *hzsA* was not immediately upstream or downstream of *hzsBC*, and few anammox metabolism genes were identified in these genomes.

### Novel nitrate/nitrite oxidoreductase genes were present in *planctomycetota*-affiliated genomes.

**Figure 5.**
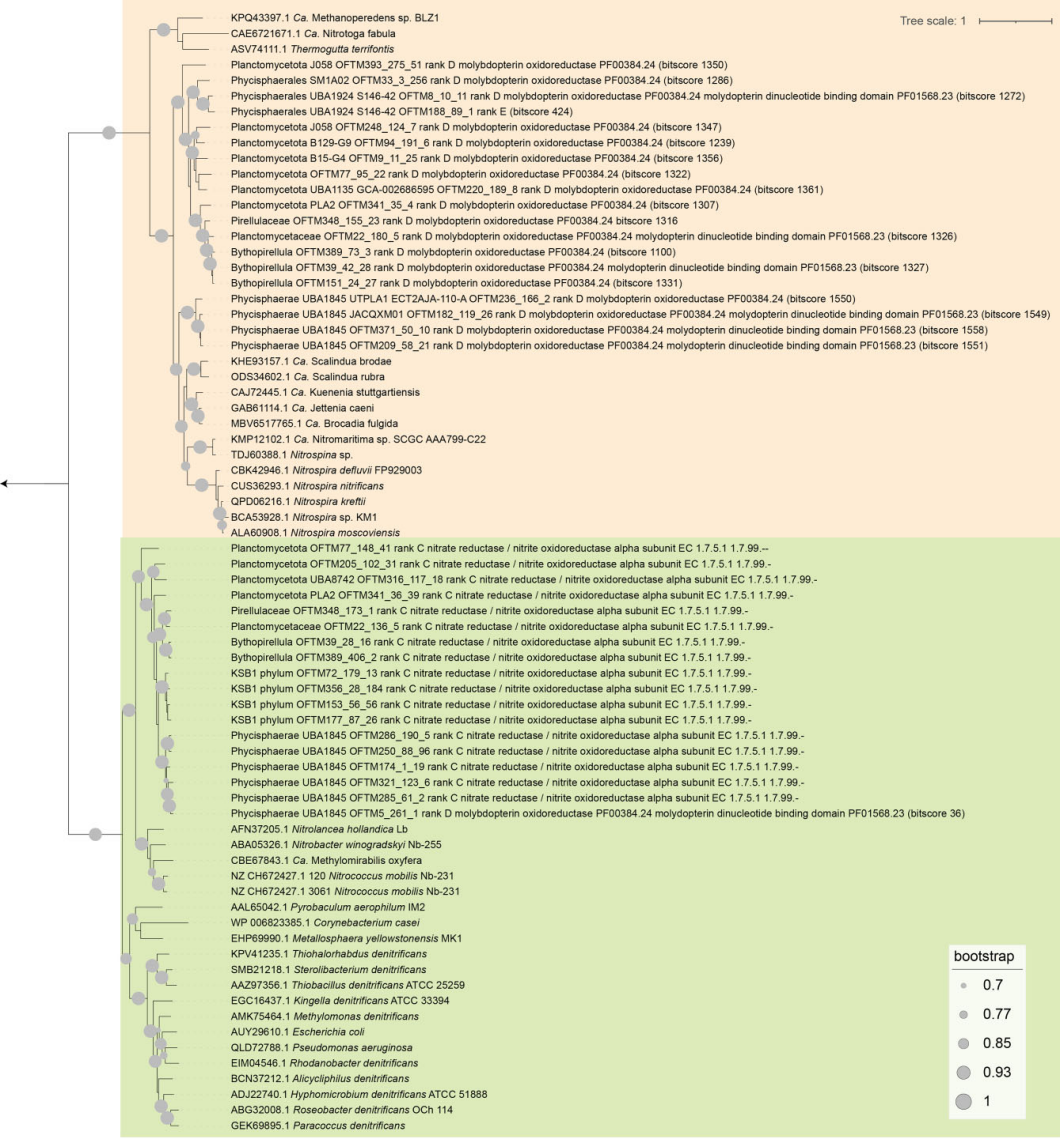
Midpoint-rooted phylogenetic tree of NarG/NxrA-encoding genes. Reference sequences were retrieved from NCBI and start with accession numbers. Other sequences were obtained from this study and are provided with DRAM annotations as well as bitscore values from blastp hits (Supplementary Table 1) to the *Ca*. Kuenenia stuttgartiensis NarG/NxrA sequence present in the tree. The two main clades are color coded in orange and green.

In total, 37 genes encoding nitrate/nitrite oxidoreductases were identified in this study (Fig. 5). Phylogenetic analyses of alpha subunit-encoding genes (NarG/NxrA) in combination with reference sequences revealed two major clades (Fig. 5). One contained reference sequences from anammox bacteria, nitrite oxidizers affiliated with *Nitrospirota, Nitrospinota*, and *Betaproteobacteria* (*Ca*. Nitrotoga fabula), the nitrate reducers *Ca*. Methanoperedens sp. BLZ1 (archaea) and *Thermogutta terrifontis* (*Plantomycetota*), and 19 sequences from this study that were poorly annotated (i.e. as ‘molybdopterin oxidoreductase’, Supplementary Table 1) but had strong blastp hits (bitscore > 1000) to *Ca*. Kuenenia stuttgartiensis NxrA, a subunit of a bidirectional nitrite oxidoreductase (Chicano et al. 2021). The second cluster contained 18 well-annotated sequences from our MAGs, reference sequences from fifteen species of nitrate reducers (lower clade in Fig. 5), and five sequences from nitrite oxidizers affiliated with *Chloroflexota* (*Nitrolancea hollandica*) and *Proteobacteria* (*Nitrobacter winogradskyi* and *Nitrococcus mobilis*) and the methane oxidizer *Ca*. Methylomirabilis oxyfera. All of these genes were part of NarGHI/NxrABC clusters in our MAGs, indicating that they likely encode novel nitrate/nitrite oxidoreductases.

While we could not assign a reaction direction (nitrite oxidation or nitrate reduction) based on our sequence analyses, we hypothesize that sequences in the first cluster (orange in Fig. 5) could represent NxrA, given the prevalence of nitrite oxidizers in this cluster and the widespread presence of genes encoding oxygen reductases, hydrogenases, and formate dehydrogenases in the 19 MAGs in this cluster (Fig. 3 and Supplementary Table 1). On the other hand, we hypothesize that 18 sequences in the second cluster (green in Fig. 5) could represent NarG, given the prevalence of nitrate reducers in this cluster. We hypothesize that these putative nitrate reducers could have a role in the observed steel fiber corrosion in the tunnel, as the activity of nitrate-reducing bacteria has been previously linked to metal corrosion, potentially via extracellular electron transfer (Miller et al. 2018, Iino et al. 2021). Out of 19 *Planctomycetota* MAGs with putative novel Nrx-type nitrite oxidoreductase-encoding genes, six MAGs (*Planctomycetota* OFTM77, *Planctomycetota* PLA2 OFTM341, *Pirellulaceae* OFTM348, *Planctomycetaceae* OFTM22, *Bythopirellula* OFTM389, and *Bythopirellula* OFTM39) also had putative Nar-type nitrate reductase-encoding genes (Fig. 3 and 5), similar to the *Chloroflexota*-affiliated nitrite oxidizer *Ca*. Nitrocaldera robusta, which harbors two types of Nar/Nxr (Spieck et al. 2020).

Most putative *nxr*-harboring MAGs had low- and/or high-affinity oxygen reductase genes and, frequently, *norB, napAB*, and *hao* (Fig. 3 and Supplementary Table 1). We infer that these MAGs could represent putatively novel nitrite oxidizers with metabolic versatility to oxidize alternative substrates coupled to a variety of terminal electron acceptors (oxygen, nitrate, nitric oxide, and ferric iron). Given that previously described nitrite oxidizers affiliate to the phyla *Proteobacteria, Chloroflexota, Nitrospirota*, and *Nitrospinota* (Daims et al. 2016b), this is the first report of putative nitrite oxidation potential in the phylum *Planctomycetota*. Genes encoding manganese, arsenite or iron oxidases were present in 12 of the 19 MAGs with putative novel *nxr* genes, indicating potential for metabolic versatility related to metal(loid) oxidation in these organisms (Fig. 3). Such potential agrees with versatility in substrate oxidation previously reported for nitrite oxidizers of the genus *Nitrospira* (Koch et al. 2015, Bayer et al. 2021) and expands the potential for metabolic versatility in putative nitrite oxidizers.

### Clusters of genes encoding proteins likely involved in nitrogen cycling were conserved across genomes.

**Figure 6.**
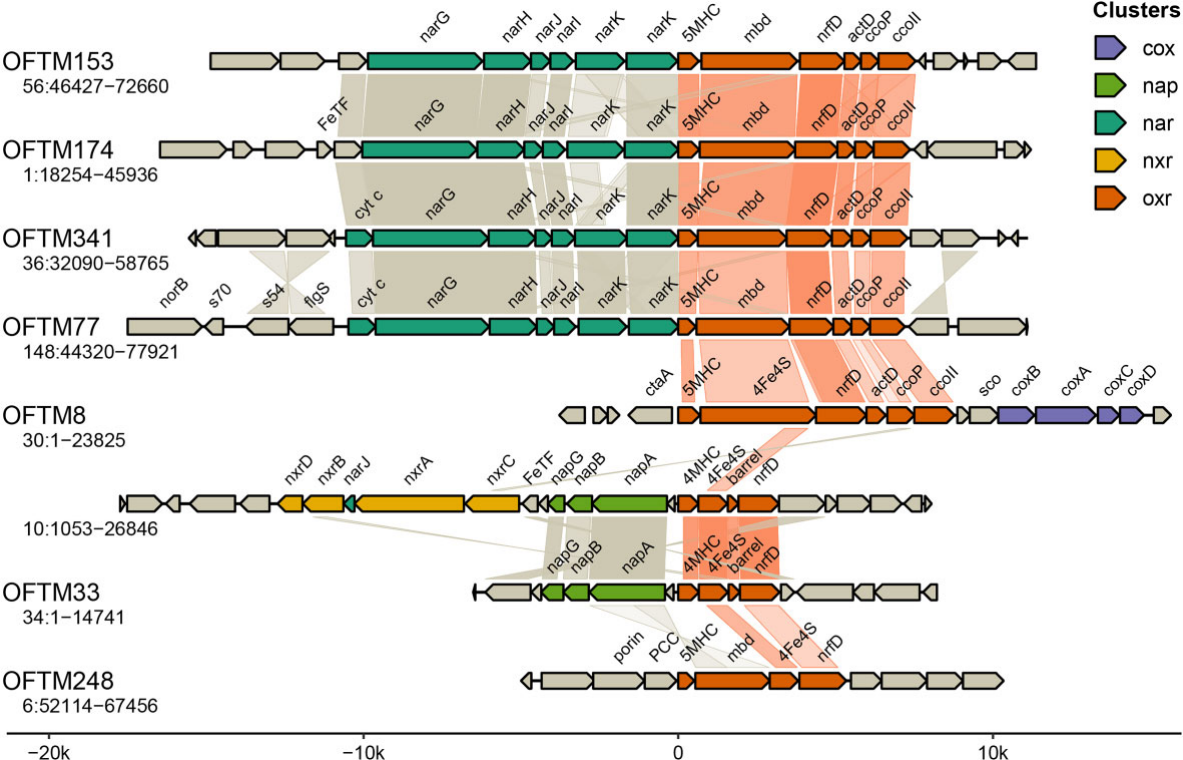
Genomic regions representative of common gene clusters potentially encoding novel ion-translocating energy-transducing membrane complexes containing an NrfD-like subunit in MAGs from this study. Genes in the molybdopterin (mbd) oxidoreductase (oxr)-containing gene cluster are color coded in orange and are abbreviated as follows: MHC, multi-heme *c*-type cytochrome (*cyt c*), with the number of heme-binding motifs indicated ahead; 4Fe4S, 4Fe-4S dicluster domain-containing protein frequently fused to the molybdopterin oxidoreductase subunit and unless indicated; nrfD, a polysulphide reductase NrfD-type putative membrane subunit; *actD*, alternative complex III transmembrane subunit D; *ccoP, cbb_3_*-type cytochrome c oxidase transmembrane subunit P; *ccoII*, transmembrane quinol:cytochrome *c* oxidoreductase quinone-binding subunit 2; barrel, Cupin domain PF07883. Genes encoding subunits of low-affinity oxygen reductases (*cox*), periplasmic nitrate reductase (*nap*), putative membrane-bound nitrate reductase (*nar*), and putative nitrite oxidoreductase (*nxr*) are color-coded in purple, green, blue, and yellow, respectively. Some genes of interest upstream or downstream of gene clusters are included: FeTF, Iron-dependent transcriptional regulator; *norB*, nitric oxide reductase; s70 or s54, regions interacting with these sigma factors; flgS, two-component system sensor kinase of the NtrC family; *ctaA*, heme *a* synthase; *sco*, synthesis of cytochrome *c* oxidase protein; porin and porin-cytochrome *c* (PCC) complexes, iron reductases.

We identified a conserved gene cluster together with putative nitrogen cycling-involved proteins across several genomes (Fig. 6). In 13 instances (Supplementary Table 1), putative Nar-encoding genes were present upstream of a six-gene cluster encoding (1) a multi-heme *c*-type cytochrome (MHC) with, most frequently, five heme-binding motifs (5MHC in Fig. 6), (2) a 4Fe-4S dicluster domain-containing protein frequently fused to a molybdopterin oxidoreductase (mbd in Fig. 6), (3) a polysulphide reductase NrfD-type putative membrane subunit, (4) an alternative complex III transmembrane subunit *actD*, (5) a *cbb_3_*-type cytochrome c oxidase transmembrane subunit *ccoP*, and (6) a transmembrane quinol:cytochrome *c* oxidoreductase quinone-binding subunit 2 (*ccoII*). This gene cluster had the architecture of an ion-translocating energy-transducing membrane complex containing an NrfD-like subunit, but did not match any previously described complexes (Calisto and Pereira 2021). Therefore, based on HHpred divergent sequence similarity analyses and on the presence of upstream putative Nar-encoding genes, we hypothesize that it could represent a novel membrane-bound NrfAH-like nitrite reductase, which converts nitrite to ammonium. Alternatively, these genes could encode for a protein part of the respiratory electron transport chain, given that, in five instances, oxygen reductase genes were downstream of the gene cluster (Fig. 6). Additionally, we identified in two MAGs (OFTM8 and OFTM33) a similar gene cluster, missing the molybdopterin oxidoreductase, *ccoP*, and *ccoII*, downstream of Nap- and putative Nxr-encoding genes, and, in one MAG (OFTM248), a similar gene cluster downstream of a porin-cytochrome *c* complex for iron reduction (Fig. 6). This further suggests a potential role for proteins encoded by this gene cluster in respiratory electron transfer.

### Potential for high metabolic versatility was detected in MAGs affiliated with the phyla KSB1 and *planctomycetota*.

We identified a variety of genes encoding proteins involved in nitrogen, oxygen, sulfur, and metal(loid) cycling in MAGs in this study (Supplementary Table 1), suggesting potential for high metabolic versatility in the microorganisms represented by these MAGs (Fig. 3). All four KSB1-affilated MAGs (OFTM72, 153, 177, and 356) had respiratory potential, with genes encoding nitrate, oxygen, and iron reductases, as well as sulfhydrogenase genes for elemental sulfur reduction to sulfide with dihydrogen gas production (Fig. 3). Only one *nosZ* gene was detected in this study, in OFTM356 (Supplementary Table 1). Additionally, the KSB1 MAGs had genes encoding arsenite and iron oxidases, hydroxylamine oxidoreductase, and genes with low sequence similarity to *hzsABC* from *Ca*. Kuenenia stuttgartiensis (Fig. 3 and Supplemental Table 1).

These results provide further evidence for the role of KSB1 bacteria in nitrogen cycling and expand the potential for high metabolic versatility in the KSB1 phylum. A recent, comprehensive analysis of 44 nonredundant, high-quality KSB1 MAGs reconstructed from groundwater, bioreactors, and marine ecosystems previously identified metabolic potential for carbohydrate and hydrocarbon degradation potentially coupled to oxygen and nitrogen respiration (*narG, nrfA, nosZ*, and *cydAB* genes) in KSB1 bacteria (Li et al. 2022). Given the low sequence similarity to canonical enzymes and the lack of an operon structure, we infer that *hzsABC*-like genes in our KSB1 MAGs are unlikely to encode a hydrazine synthase. Instead, we hypothesize that the prevalence of *hzs*-like genes with low sequence similarity to canonical anammox genes in MAGs from this study indicates that hydrazine synthase-like enzymes may comprise a broader, widespread enzymatic family with potential for activity with alternative substrates.

While all MAGs in our study had potential for nitrogen cycling, 26 of 33 MAGs also had potential for metal(loid) cycling, suggesting that bacteria represented by these genomes might couple these reactions. Of 29 *Planctomycetota* MAGs, 15 had genes encoding manganese oxidases, 3 encoding arsenite oxidases, and 5 encoding iron oxidases, which might be coupled to nitrate or oxygen respiration in these microorganisms (Fig. 3). Additionally, iron reduction potential was detected in four *Planctomycetota* MAGs. A coupling of iron oxidation and nitrate reduction has been observed before in the family *Gallionellaceae* (He et al. 2016) and the DTB120 candidate phylum (McAllister et al. 2021), and this study suggests that it might also occur in *Planctomycetota*.

To our knowledge, this is the first report of potential for manganese and iron cycling in nonanammox bacteria in the phylum *Planctomycetota* (Wiegand, Jogler and Jogler 2018, Kappler et al. 2021). However, 16S rRNA gene analyses of microbial mats from an iron-rich thermal spring (Selvarajan et al. 2018), deep sea iron hydroxide deposits (Storesund and Øvreås 2013), and metalliferous deposits from hydrothermal vents (Storesund et al. 2018) have previously identified abundant *Planctomycetota* groups, including *Ca*. Brocadiales and *Phycisphaerae* UBA1845. Additionally, the *Planctomycetota* bacterium *Bythoypirellula goksoyri* was isolated on organic carbon sources under oxic conditions from deep sea iron hydroxide deposits (Storesund and Øvreås 2013). In our study, one of three MAGs affiliated with *Bythoypirellula* had a Cyc2-encoding gene, indicating potential for iron oxidation in these microorganisms, which aligns with their isolation source. These results expand the phylogenetic diversity of microorganisms putatively involved in metal cycling. Together with *Zetaproteobacteria*, which has been previously detected in Oslofjord tunnel biofilms (Karačić et al. 2018), these bacteria affiliated with *Planctomycetota* and KSB1 could contribute to iron oxidation in the Oslofjord tunnel, potentially contributing to steel fiber corrosion. Finally, such microorganisms could play a role in microbially-induced corrosion of built infrastructure in other marine environments.

## Conclusions

The deep biosphere remains largely unexplored due to sampling costs and challenges. However, microbial communities in these ecosystems may harbor untapped potential for novel biogeochemical reactions in the nitrogen cycle and biotechnological applications. This study took advantage of samples from a unique, oxygenated deep marine ecosystem, the Oslofjord tunnel, to explore the potential for such novel metabolic capabilities in microorganisms enriched in concrete-degrading biofilms. We identified potential for nitrogen and metal cycling in novel taxa within the phyla *Planctomycetota* and KSB1, hypothesizing that these microorganisms might be previously unrecognized anammox, nitrite-oxidizing, and nitrogen- and metal-cycling bacteria. These results expand the known diversity of microorganisms putatively involved in these important biogeochemical reactions, and contribute to our understanding of potential biofilm impacts on built infrastructure.

## Supplementary Material

fnad049_Supplemental_File
